# In Vitro Culture of Human Dermal Fibroblasts on Novel Electrospun Polylactic Acid Fiber Scaffolds Loaded with Encapsulated Polyepicatechin Physical Gels

**DOI:** 10.3390/gels10090601

**Published:** 2024-09-20

**Authors:** Eliza Miranda-Buendia, Gertrudis H. González-Gómez, Alfredo Maciel-Cerda, Maykel González-Torres

**Affiliations:** 1 Facultad de Ciencias, Universidad Nacional Autónoma de México, Ciudad de México 04510, Mexico; emirandabuendia@outlook.es (E.M.-B.); hortecgg@ciencias.unam.mx (G.H.G.-G.); 2 Instituto de Investigaciones en Materiales, Universidad Nacional Autónoma de México, Ciudad de México 04510, Mexico; 3 Conahcyt & Laboratorio de Biotecnología, Instituto Nacional de Rehabilitación “Luis Guillermo Ibarra”, Ciudad de México 14389, Mexico

**Keywords:** epicatechin, polyepicatechin, scaffolds, electrospun, human dermal fibroblast, hydrogel

## Abstract

Polyepicatechin (PEC) in a hydrogel has previously shown promise in enhancing physiological properties and scaffold preparation. However, it remains unclear whether PEC-based fibers can be applied in skin tissue engineering (STE). This study aimed to synthesize and characterize electrospun PEC physical gels and polylactic acid (PLA) scaffolds (PLA_loaded_PEC_sub_) for potential use as constructs with human dermal fibroblasts (HDFs). PEC was produced through enzymatic polymerization, as confirmed by Fourier transform infrared (FTIR) spectroscopy. Scanning electron microscopy (SEM) demonstrated the feasibility of producing PLA_loaded_PEC_sub_ by electrospinning. The metabolic activity and viability of HDFs cocultured with the scaffolds indicate that PLA_loaded_PEC_sub_ is promising for the use of STE.

## 1. Introduction

Epicatechin (EC), a common flavonoid, has antioxidant properties due to its phenolic hydroxyl groups [1]. Studies suggest enhanced physiological benefits and a longer useful life of polymeric forms of EC [2]. Enzymatic polymerization, especially with laccases, is crucial for versatility and cost effectiveness [3]. Recently, hydroxypropyl-β-cyclodextrin (HP-β-CD)/epicatechin (EC) clathrate compounds were rapidly synthesized via an ultrasound-mediated method, and polycaprolactone (PCL)/locust bean gum nanofibers loaded with these clathrate compounds were fabricated via electrostatic spinning (ELS) for potential fruit packaging applications. Infrared spectrum and crystal-type analyses confirmed the successful preparation of the clathrate compounds. The inclusion of clathrate compounds resulted in an increase in the fiber diameter from 553.43 to 1273.47 nm and the formation of hydrogen bonds between clathrate compounds and fibrous membranes, which improved the thermal stability, reduced the crystallinity, and improved the hydrophilicity and gas permeability. The fibrous membranes exhibited a sustained release of EC over 240 h, maintaining the activity of EC and demonstrating significant bacteriostatic properties both in vitro and in vivo. These findings suggest that the antibacterial fibrous membranes developed in this work have promising applications for fruit packaging [4]. Furthermore, another recent study described the development of biodegradable and environmentally friendly packaging films through the preparation of active packaging bilayer films. The films were created by combining chitosan-fish gelatin (CS-FG) with varying concentrations of epigallocatechin-3-gallate (EGCG) on the top layer of polylactic acid (PLA) film via either solvent casting (SC-films) or electrospinning (ES-films). The incorporation of EGCG (0.5–2%) significantly improved the tensile strength, film thickness, water vapor barrier properties, seal strength, and antioxidant activities (*p* < 0.05) while reducing the swelling capacity and light transmittance (*p* < 0.05) [5]. Similarly, electrospun catechin-loaded nanofibers were fabricated and characterized for use in the fortification of milk. In this research, the challenges associated with the use of catechins as nutraceuticals in food were addressed by encapsulating them within zein-based nanofibers through electrospinning. The electrospinning parameters, including zein concentrations (15%, 18%, and 21% *w*/*w*), applied voltages (16, 20, and 24 kV), and feed rates (0.5 and 1.0 mL/h), were optimized using the Taguchi L18 design. The optimal conditions resulted in nanofibers with a high encapsulation efficiency of 92.8% and an average diameter of 95.2 nm. Specifically, at a concentration of 18% zein, a feed rate of 0.5 mL/h, and 20 kV, the resulting nanofibers were clean, were bead-free, were cylindrical, and had a nonporous structure. The nanofibers were characterized, revealing a hydrodynamic diameter of 172.3 nm, a zeta potential of −26.3 mV, and a polydispersity index of 0.15. Catechin encapsulation was confirmed by Fourier transform infrared (FTIR) spectroscopy and X-ray diffraction. Catechins are released from nanofibers in a controlled and sustained manner, maintaining their antioxidant properties. Furthermore, the incorporation of these nanofibers into milk did not alter their physicochemical and sensory qualities [6]. The incorporation of phenolic compounds into electrospun nanofibers has been shown to function effectively as nanovehicles [7]. Although the use of polyphenols loaded onto electrospun nanofibers has been investigated in tissue engineering (TE), not much has been done specifically with epicatechin in this area [8].

Poly(catechins) can be synthesized from catechins via enzymatic chemistry. A unique enzymatic approach has been reported to synthesize water-soluble poly(catechins) with enhanced stability and potent antiproliferative effects in human cancer cells in vitro [9]. Various stereoisomers of catechin [(+), (−), (±)] and (−)-epicatechin have been biocatalytically polymerized via horseradish peroxidase (HRP) in ethanol/buffer mixtures. This one-pot biocatalytic polymerization, conducted under ambient conditions, yielded water-soluble poly(catechins). These synthesized PECs have been tested for their growth inhibitory properties in a variety of normal and cancerous human epithelial cell lines. Compared with monomers, poly(catechins) exhibited statistically significant growth inhibitory effects; specifically, they inhibited the growth of breast, colorectal, and esophageal cancer cells but had little effect on normal epithelial cell growth, thus achieving a high therapeutic ratio.

PEC is present in fruits such as apples, grapes, and kiwis or in extracts such as *Stryphnodendron* astringents and *Abarema cochliacarpa* [10,11,12,13,14]; however, PEC is composed of a few monomers; for example, PEC in these reports is an oligomer; also, PEC can be found as procyanidin. Procyanidin B2 inhibits IL-17, TNF-α, and IL-1β, which are involved in the development of inflammation [15]. On the other hand, nicotine has been shown to suppress the expression of DHFR and GCH1, which are enzyme intermediaries that synthesize BH4, and procyanidin increases the expression levels of these enzymes [16]. According to Ares et al., oligomeric procyanidins show antimicrobial and enzyme inhibitory activities, and trimeric and polymeric procyanidins are more inhibitory than dimeric procyanidins [17].

During the browning of the pericarp in some fruits, such as litchi, apple, and sweet cherry, the formation of brown products corresponds to the oxidation of flavanols to quinones. In addition, the oxidative polymerization of flavonoids in vitro with laccase has been reported. To discern the underlying mechanism, laccase-generated A- and B-type polymers (brown products) were generated from laccases using EC and catechin as substrates [18,19].

Despite the potential of polyepicatechin (PEC) as a bioactive component, its use in TE is underexplored. This gap highlights the need for further research to determine the full potential of PEC-based materials [20]. Furthermore, during our electrospinning research, we consistently observed that certain polymer solutions tend to form physical gels, a behavior different from the precipitation observed in ethylene–vinyl alcohol copolymer (EVOH) systems. Specifically, poly(vinylidene fluoride) (PVDF) solutions in dimethyl formamide (DMF) often turn into physical gels after cooling to room temperature for several hours, especially when the polymer concentration is high [21].

The use of catechins as gels in medicine has been explored. An earlier study emphasized the importance of controlling microbial growth and biota after oral cleaning to prevent disease onset. Catechin has been highlighted for its potential periodontal applications. When combined with a gel (catechin gel), its antimicrobial activity and antioxidation properties are prolonged. Catechin gel inhibited the growth of Actinomyces, periodontopathic bacteria, and Candida strains but not the oral *Streptococci* essential to the normal oral flora. In contrast, commercially available moisture gels with antimicrobial components inhibited all strains tested, including oral streptococci. This demonstrated the selective antimicrobial activity of catechin because of hydrogen peroxide production. This study reviewed previous work on catechins and suggested their potential application in the prevention of dental caries and periodontal disease [22]. Recently, the extraction and isolation of catechins from various plant sources via different analytical techniques were reviewed. Catechins, known for their diverse pharmacological properties, including anti-inflammatory, neuroprotective, antioxidant, antibacterial, anticancer, and antiviral activities, were analyzed in this review. Various formulations that incorporate catechins were reported, highlighting their multiple uses. The review also covered synthetic and biosynthetic procedures for catechins, as well as clinical trials and patents related to them. The significant outcomes and modes of action of the pharmacological activities of catechin are discussed. The need for more research to resolve bioavailability and unclear modes of action has emphasized the need to develop effective catechin-based therapies [23].

In the field of TE, an adhesive hydrogel dressing that incorporates antibacterial, anti-inflammatory, and antioxidant properties for wound repair was recently developed. The dressing was composed of polyethylene glycol diacrylate (PEGDA), chitosan (CS), and catechin-loaded mesoporous silicon dioxide (CMSN). The hydrogel adhered tightly to tissues through the topological adhesion of CS macromolecules, whereas CMSN slowly released catechin at the wound site. The intermolecular interactions and hydrogen bonding between the CMSN and PEGDA/CS hydrogels enhanced the mechanical stability of the dressing, enabling effective closure and repair of wounds, even in areas with frequent movement. Furthermore, red light therapy has been used to further promote wound healing by improving blood circulation, improving wound oxygenation, and stimulating collagen synthesis. This combination has offered a promising approach to the rapid repair of wounds in motorial areas [24].

Recently, catechin was incorporated into a fibrous structure composed of gelatin and PLA to produce an electrospun material intended for wound dressings by adding L929 fibroblasts. The addition of this flavonoid increased fibroblast viability, but the extract did not have toxic effects [25]. On the other hand, a hydrogel in which polyphenols are coordinated with Fe^3+^ and vinyl monomers was developed in an adhesive experiment on human skin and showed no adverse effects [26]. An important question is whether PEC can exhibit these biological features when combined with PLA. The significance of this work lies in the exploration of PECs within electrospun scaffolds for in vitro cell culture.

The hypothesis is that PLA_loaded_PEC_sub_ combined with HDFs will have potential applications in regenerative medicine. We suggest that the unique properties of PECs, such as covalent and noncovalent interactions, positively influence the culture of HDFs, contributing to the field of STE [27,28,29]. This study aimed to synthesize and characterize these scaffolds while evaluating the in vitro culture of HDFs. To the best of our knowledge, there are no reports of PLA_loaded_PEC_sub_ constructs that incorporate fibroblasts.

## 2. Results and Discussion

### 2.1. Composition by ATR-FTIR

**Figure 1:**
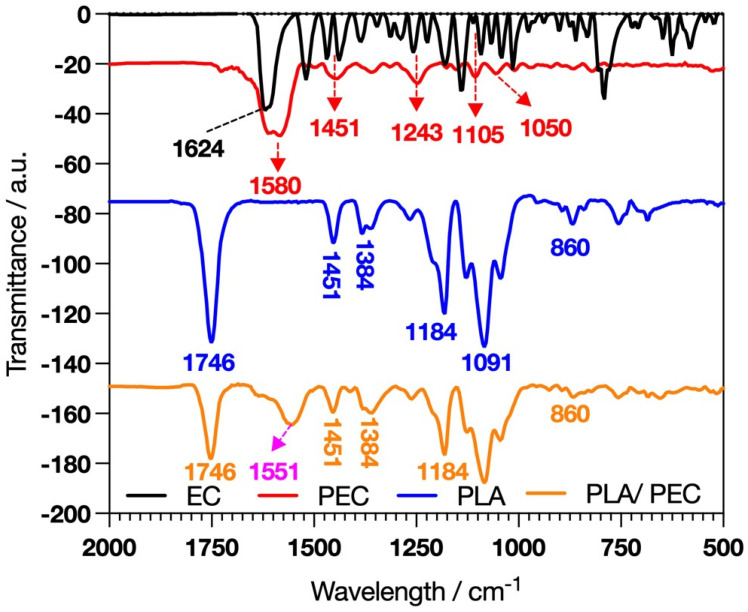
FTIR spectral analysis showing the characteristic profiles of EC, PEC, PLA, and the composite PLA_loaded_PECsub.

The polymerization we developed was carried out as described by Gonçalves et al. [30], with the key difference being the use of flavanols. Giménez et al. reported that catechin reacts faster than EC because EC is less planar than catechin; however, laccase requires similar concentrations of catechin and EC to achieve the kinetic behavior of Vmax [31]. FTIR analysis was developed to characterize the synthesis of PEC and the elaboration of PLA_loaded_PEC_sub_ scaffolds. EC and PEC (Figure 1) revealed distinctive absorption bands, such as C=O stretching at 1624 cm^−1^ [32].

PEC exhibited pronounced hydrogen-bonded hydroxyl groups in the 3800–3000 cm^−1^ range, suggesting that laccase-catalyzed oxidative reactions occurred [33,34,35]. Differences in the absorption bands at 1600–1000 cm^−1^ indicate more rigid functional groups in PEC than in EC [33]. These spectral characteristics are significant because they provide insight into the potential for crosslinking PECs with proteins such as gelatin or collagen to form a hydrogel. These hydrogels can leverage the bioactive properties of PECs, combined with the structural and mechanical benefits of protein crosslinking, to create materials suitable for TE and regenerative medicine applications.

In addition, the development of hybrid bioactive nanofibers that combine PLA and gelatin through sol–gel crosslinking and electrospinning further demonstrates the versatility and potential of these materials [36]. The combination of PEC with gelatin or collagen in a hydrogel matrix or PLA in a fibrous scaffold exemplifies the innovative approaches explored to create multifunctional biomaterials.

The success of the PLA_loaded_PEC_sub_ scaffolds was indicated by the presence of a broad band in the range of 3800–3000 cm^−1^, suggesting interactions through hydrogen bonding of PEC hydroxyl groups with the PLA fibers [37]. In this framework, the intensity of the PLA band attributed to C=O stretching at 1746 cm^−1^ decreases [38,39]. Furthermore, the band at 1580 cm^−1^ in PEC, due to the aromatic stretching of C=C-C, shifted to 1553 cm^−1^, suggesting an interaction between PEC and PLA [40].

### 2.2. Contact Angle

**Figure 2:**
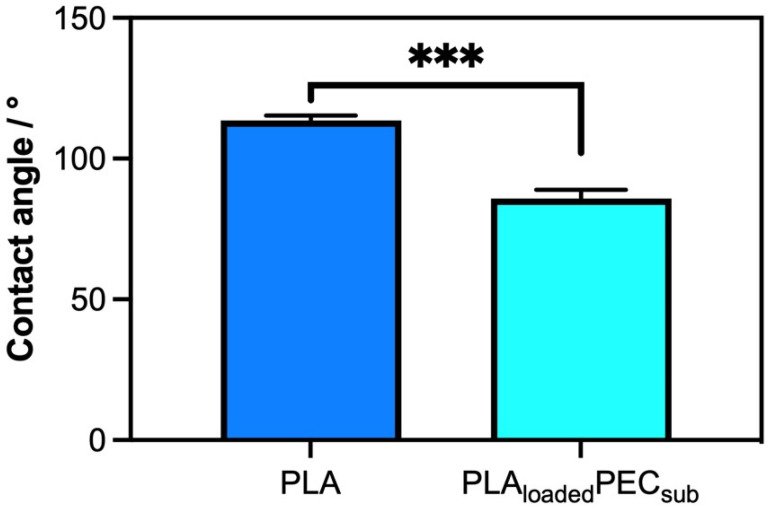
Water contact angle measurements of PLA and PLA_loaded_PEC_sub_ represented in degrees. *** *p* ≤ 0.001.

Figure 2 shows a comparison of the surface properties, specifically hydrophobicity, of PLA- and PLA_loaded_PEC_sub_. The scaffold or PLA presented an angle contact of 113.6° ± 1.76°, confirming its hydrophobic properties, as reported in the literature [41,42]. Apparently, the PEC decreased the contact angle to 85.81° ± 81° ± 3.14°, indicating an affinity for water. This could be explained by the fact that the hydroxyl groups of PLA_loaded_PEC_sub_ improve the water absorption of the surface [42].

### 2.3. Antioxidant Activity

**Figure 3:**
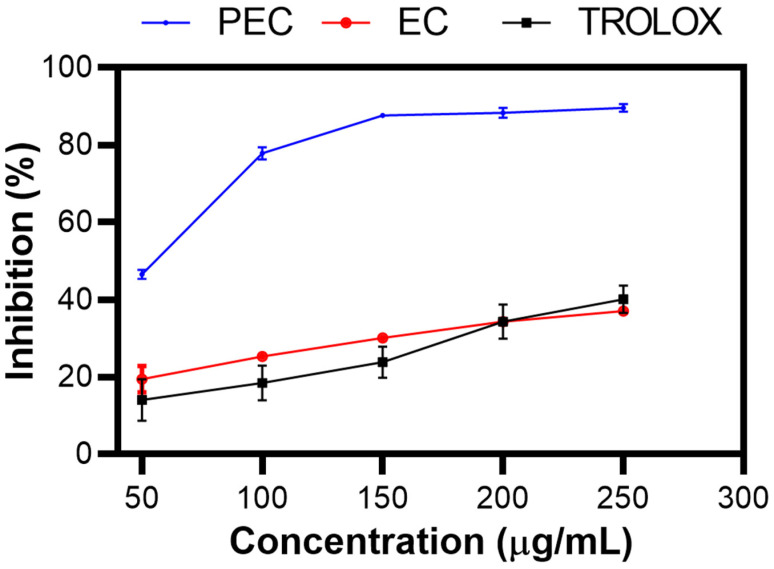
Inhibition (%) of PEC, EC, and TROLOX. ABTS inhibition assay.

Figure 3 shows the % inhibition of PEC, EC, and TROLOX as a control, as determined by the ABTS assay [43]. As the concentration increased, the percentage inhibition increased, indicating dose-dependent antioxidant activity. This trend was clearly observed in the experimental results, showing that PEC had a greater percentage of inhibition than did EC, as Zhang et al. reported [44]. This finding suggests that the polymerized structure of PEC enhances its ability to neutralize free radicals more effectively than its monomeric form. However, a discrepancy was observed in the study by Chen X et al., who reported different inhibition percentages under similar conditions [45].

The strong antioxidant activity of PEC is significant because of the well-known association between reactive oxygen species (ROS) and various types of skin damage. ROS are highly reactive molecules that can cause oxidative stress, leading to cell damage, aging, and various skin diseases. The development of a substrate with potent antioxidant activity, such as PEC, can help mitigate these harmful effects by scavenging ROS and protecting skin cells from oxidative damage. This protective effect is crucial for skin care and wound healing applications, where maintaining a healthy cellular environment is essential for proper tissue repair and regeneration [46,47,48].

Furthermore, the ability of PEC to inhibit ROS not only underscores its antioxidant potential but also suggests its potential for antibacterial and anti-inflammatory activities. ROS are involved in the inflammatory response and bacterial proliferation. By reducing ROS levels, PEC can help reduce inflammation and inhibit the growth of bacteria, making it a valuable component in the development of new therapeutic agents for the treatment of skin infections and inflammatory conditions [49].

### 2.4. Fiber Morphology

**Figure 4:**
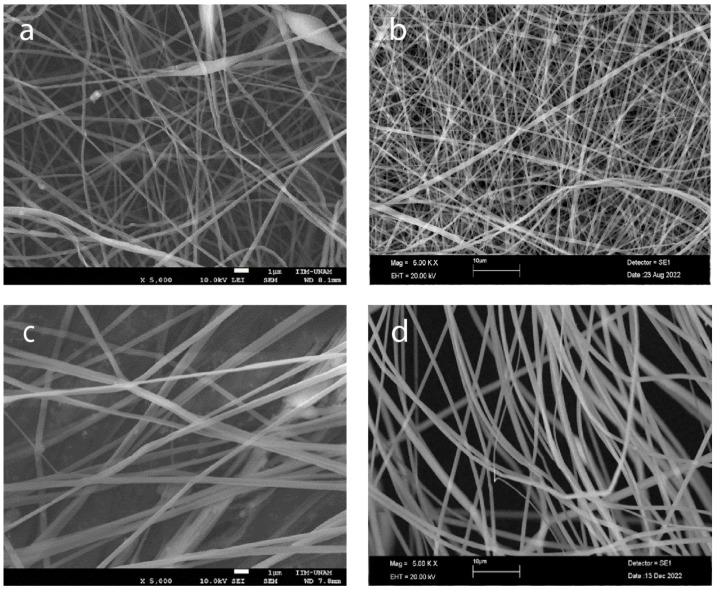
Morphology of electrospun scaffolds of (**a**) PLA 6% *w*/*v*, (**b**) PLA 13%, (**c**) PLA 16%, and (**d**) PLA 20% (scale bars 1 μm, 10 μm, 1 μm, and 10 μm, respectively).

**Figure 5:**
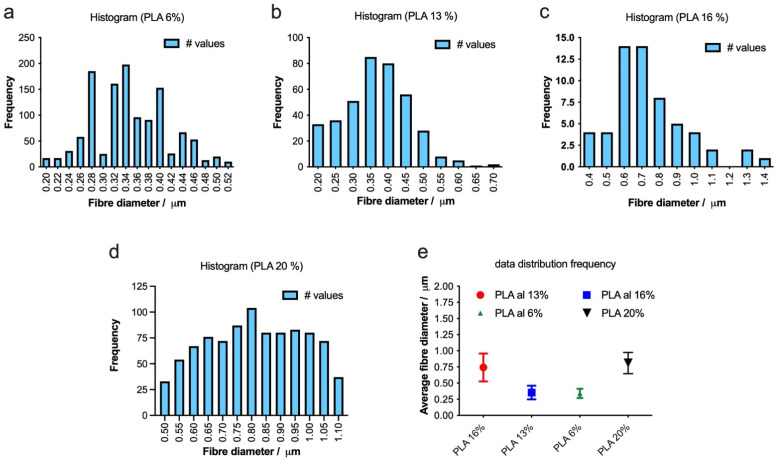
(**a**–**d**) Histograms showing the distribution of fiber diameters for electrospun scaffolds with varying PLA concentrations (6%, 13%, 16% and 20% *w*/*v*). The bar graphs illustrate the frequency of fiber diameters, and the summary plot (**e**) displays the average fiber diameters for each PLA concentration.

To optimize the production of defect-free fibers, we employed electrospinning to fabricate fibers with varying concentrations of PLA 6, 13, 16, and 20%, as depicted in Figure 4. As the PLA concentration increases, the average fiber diameter also increases, and the distribution of the diameter becomes narrower, indicating more uniform fiber formation (Figure 5a–e). The highest concentration of PLA at 20% *w*/*v* produced the most consistent fibers, both in terms of diameter and distribution (Figure 4d and Figure 5d). This suggests that increasing the PLA concentration in the electrospinning solution improved the fiber uniformity and consistency, resulting in a tighter distribution of fiber diameters.

The challenge was to load PECs onto the fibers. The advantage of using two separate nozzles [50,51,52], with one syringe containing the PEC solution and the other containing the PLA solution, was that it eliminated the need to find a common solvent for both PLA and PEC. This procedure was unnecessary because PEC does not dissolve in hexafluoroisopropanol (HFIP) to obtain the encapsulated PEC, and a low-concentration solution and a low-molecular-weight polymer were necessary [53,54].

**Figure 6:**
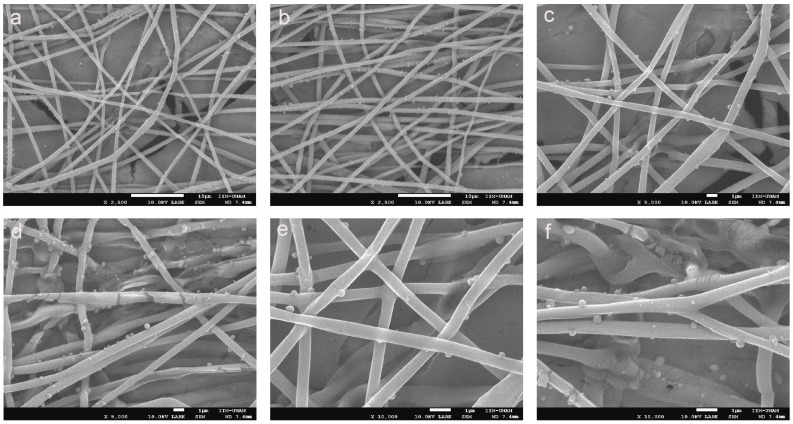
SEM images of (**a**–**c**) PLA and (**d**–**f**) PLA_loaded_PEC_sub_ (scale bars = 10 μm, 10 μm, 1 μm, 1 μm, 1 μm, and 1 μm, respectively).

Figure 6a–f show PLA_loade_dPEC_sub_, which has a highly uniform and well-formed fibrous structure, indicative of a successful electrospinning process, which has a highly uniform and well-formed fibrous structure, indicative of a successful electrospinning process [55]. The fibers appear smooth and continuous, with a consistent diameter throughout the sample. This uniformity is crucial for ensuring predictable mechanical properties and reliable performance in biomedical applications [56]. Additionally, Figure 6d–f of PLA_loaded_PEC_sub_ reveal small spherical particles distributed on the fiber surface, which is probably indicative of the incorporated PEC. These particles suggest that PEC is effectively integrated into the PLA matrix, possibly through physical entrapment or chemical bonding during the electrospinning process. The presence of these particles can enhance scaffold bioactivity by providing antioxidant properties, which are essential for protecting cells from oxidative stress in tissue engineering applications.

**Figure 7:**
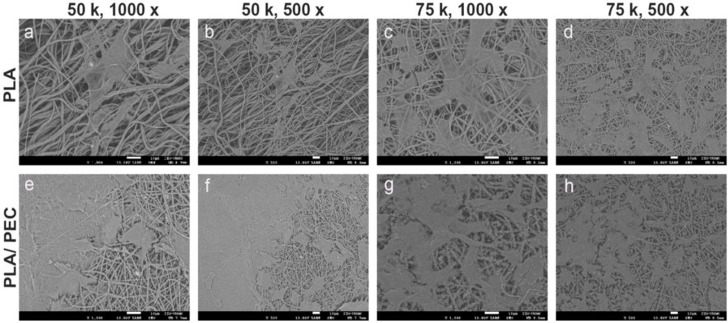
SEM images showing the microstructure of electrospun scaffolds. (**a**–**d**) Pure PLA scaffolds, and (**e**–**h**) PLA/PEC scaffolds cocultured with HDFs, at different magnifications (1000× and 500×). The images highlight the fiber morphology and surface interactions, with a scale bar of 10 µm.

The dense, interconnected fiber network shown in Figure 4 closely mimics the extracellular matrix, providing an ideal environment for cell attachment, proliferation, and migration. This structural similarity to the natural extracellular matrix is critical for promoting effective tissue regeneration. The smooth surface of the fibers, combined with the subtle texture provided by the PEC particles, is likely to enhance the cell-scaffold interactions, promoting improved cell adhesion and growth, as shown in Figure 7a compared with Figure 7c [55]. Moreover, the minimal presence of defects, such as beads or irregularities, further underscores the quality of the electrospinning process and the robustness of the resulting scaffold. Together, these characteristics suggest that PLA_loaded_PEC_sub_ scaffolds are well suited for STE and other regenerative medicine applications, offering a multifunctional platform that combines structural support with enhanced bioactivity and antioxidant protection.

Figure 7 shows SEM images at 1000× and 500× magnification of the PLA- and PLA_loaded_ PEC_sub_ scaffolds cultured with fibroblasts at cell densities of 50,000 cells/cm^2^ and 75,000 cells/cm^2^, respectively. HDFs tended to cover the surface more extensively on PLA_loaded_PEC_sub_ scaffolds than on the PLA scaffolds. The cells exhibited a typical fibroblast morphology on both scaffolds, characterized by an extended, starshaped, and elongated cellular structure. There are no reports in the literature on the effects of cell culture on PLA_loaded_PEC_sub_ scaffolds. Nevertheless, HDFs seeded on PLA scaffolds obtained through electrospinning demonstrate superior spreading and adhesion compared with murine fibroblasts from the BALB/c 3T3 lineage seeded on scaffolds of the same biomaterial [57]. Another study showing the ability of PLA as a scaffold benefit for wound healing in vitro using fibroblasts led us to expect that PEC improved these properties [58].

### 2.5. Tensile Test

**Figure 8:**
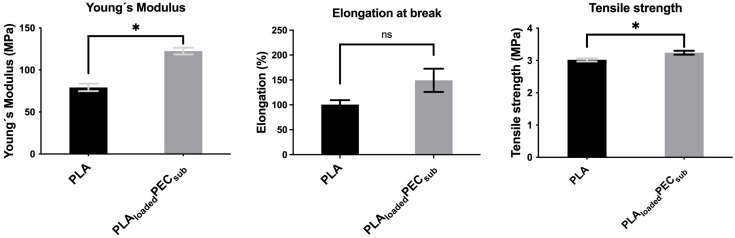
Tensile test of the PLA- and PLA_loaded_PEC_sub_ scaffolds. Not significant (ns) at *p* > 0.05, and * *p* ≤ 0.05.

Figure 8 shows the Young’s modulus, elongation at break, and tensile strength of the PLA and PLAloadedPECsub scaffolds. PEC increases the Young’s modulus and tensile strength, suggesting the effective interaction of hydrogen bonds between PEC and PLA, as mentioned in the FTIR spectra above. The difference in elongation at break was not significant.

### 2.6. Thermal Analysis

**Figure 9:**
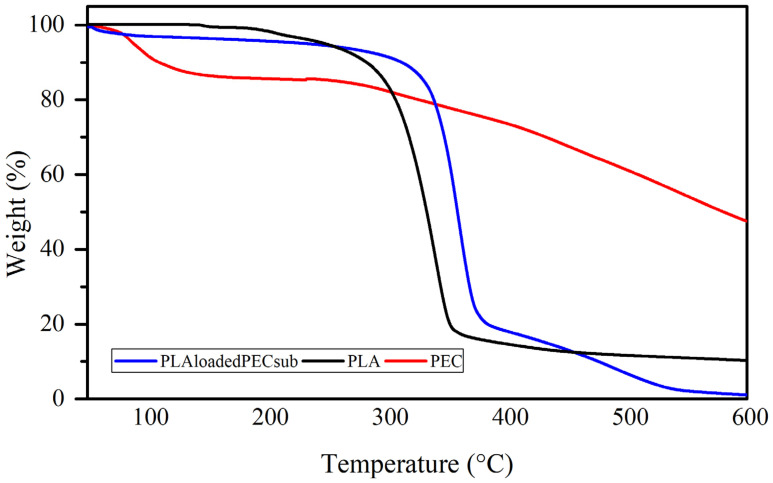
Weight loss profiles of the PEC, PLA, and PLA_loaded_PEC_sub_ scaffolds.

The weight loss profiles of the PEC, PLA, and PL-loaded PEC scaffolds determined by TGA are depicted in Figure 9. The PEC curve has two weight loss steps: the first step has a maximum rate temperature of 86 °C, with mass losses ranging from 100 to 87%; this could be partly due to the evaporation of water. The second step, with a maximum rate temperature of 448 °C and mass losses ranging from 87 to 40%, is attributed to the decomposition of PEC. The curve of the PEC does not degrade completely, indicating good resistance to degradation [59,60]. The curve of the PLA_loaded_PEC_sub_ scaffold shows a first step with a maximum rate temperature of 56 °C, with mass losses ranging from 100 to 97% influenced by PEC particles. The second step has a maximum rate temperature of 358 °C, with mass losses ranging from 97 to 18%, and a third step from 18 to 2% weight, with a maximum rate temperature of 501 °C. In the PLA curve, three mass losses occur, one starting at 127 °C and ending at 182 °C, with a maximum temperature ratio of 147 °C; the next mass loss is 3%, with a maximum temperature ratio of 205 °C; and the third step is 85%, with a maximum temperature ratio of 339 °C.

**Figure 10:**
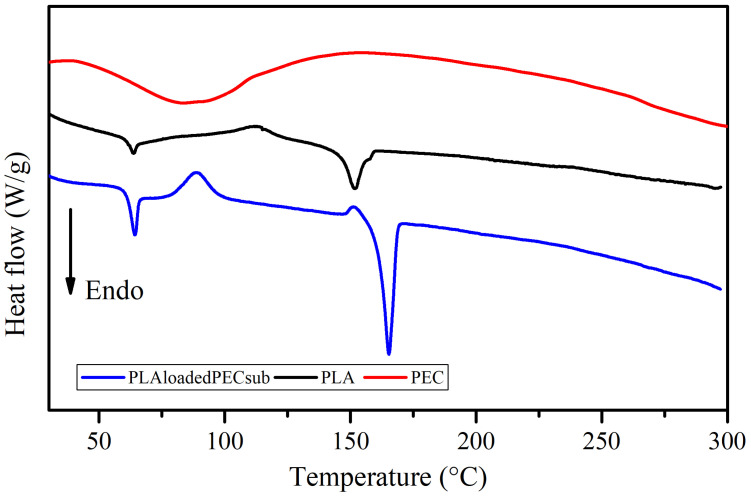
DSC thermograms of the PEC, PLA, and PLA_loaded_PEC_sub_ scaffolds.

The DSC profiles of the PEC, PLA, and PLA_loaded_PEC_sub_ scaffolds are shown in Figure 10. There is a broad endothermic peak in the curve corresponding to PEC in the range of 60 to 100 °C. Water evaporation may explain this, which is consistent with the TGA profile. The PLA_loaded_PEC_sub_ scaffold was characterized by a thin endothermic peak with a maximum melting rate of 165 °C, which is greater than the maximum temperature of the PLA scaffold of 152 °C. This outcome could result from an increase in flexible chain molecules due to the addition of PEC. Moreover, further studies are needed to assess this phenomenon [61,62]. In addition, the melting of the PLA_loaded_PEC_sub_ scaffold started at 143 °C and ended at 173 °C, corresponding to a broad range of temperatures, resulting in a mixture of polymers (Brittain and Bruce [63]). The addition of PEC led to significant changes in thermal behavior, particularly in the glass transition temperature (Tg), which remains at 64 °C but shows a more pronounced signal in the PLA_loaded_PEC_sub_ scaffold than in the PLA scaffold. Notably, a crystallization peak appears at 89 °C in the PLA_loaded_PEC_sub_ scaffold, whereas the PLA scaffold shows a less pronounced crystallization peak at 113 °C. Additionally, the enthalpy of fusion for the PLA_loaded_PEC_sub_ scaffolds increased to 48 J/g, whereas it increased to 24 J/g for PLA, demonstrating enhanced thermal stability.

### 2.7. Cell Culture

**Figure 11:**
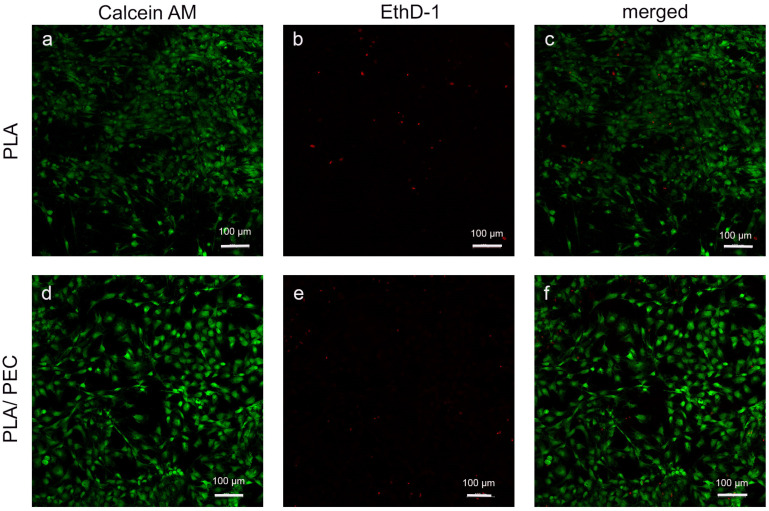
(**a**–**f**) Fluorescence-stained images depict HDFs in PLA- and PLA_loaded_PEC_sub_ after 24 h of cultivation (scale bar: 100 μm). The first row shows the calcein AM channel, the second row shows the EthD-1 channel, and the final row shows the merged calcein AM/EthD-1 channel.

Studies of the calcein/ethidium homodimer indicated that HDFs cultured for 24 h on biomaterials were metabolically active and had relatively high viability. A greater number of dead cells (highlighted in red) was observed on the PLA scaffold than on the PLA_loaded_PEC_sub_ scaffold (Figure 11).

**Figure 12:**
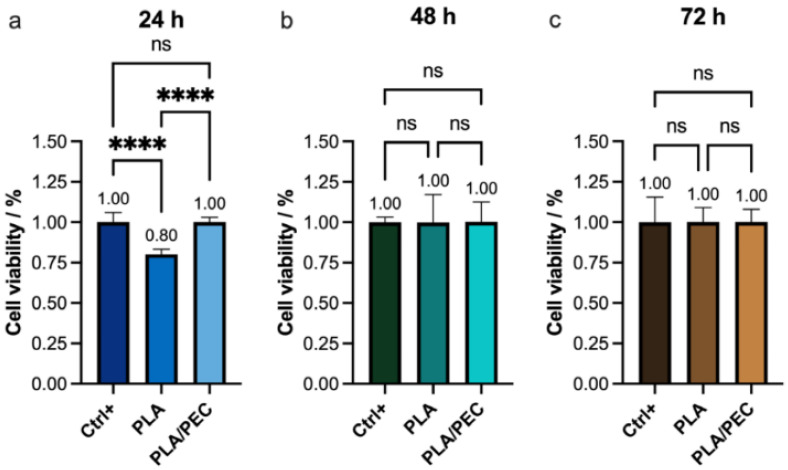
(**a**–**c**) CCK-8 assay showing the viability of the HDF monolayer (Ctrl+) and the monolayer after (**a**) 24 h, (**b**) 48 h, and (**c**) 72 h of exposure to PLA and PLA_loaded_PEC_sub_. The absence of lines and symbols above the bars indicates statistical non-significance (ns) in the experiment (*p* > 0.05), whereas **** denotes statistical significance at *p* < 0.001 (technique: one-way analysis of variance (ANOVA)).

These findings were further corroborated by proliferation and cytotoxicity (CCK-8) assays, which revealed a significant decrease in viability in the PLA group (80%) compared with the PLA_loaded_PEC_sub_ scaffold and monolayer groups (Figure 12). This result is consistent with the findings reported by Scarpini et al. [57]. The results did not reveal significant differences in cell viability after 48 or 72 h of culture. Previous studies have indicated that increasing PEC concentrations have a negligible effect on tumor cell viability [64,65]. These results suggest that the incorporation of PEC molecules into PLA during electrospinning positively influences their molecular interactions with fibroblasts. A potential limitation of this study is that it focused on in vitro evaluations.

The greater cell viability observed at 24 h with PLA_loaded_PEC_sub_ than with PLA alone suggests that the initial interaction between the cells and the PLA_loaded_PEC_sub_ scaffold is more favorable, likely due to the bioactive properties of PEC, which may enhance cell attachment and proliferation. However, the lack of a significant difference at 48 and 72 h suggests that the initial increase in cell viability provided by PEC does not translate into long-term differences under the conditions tested. This could indicate that while PEC has an immediate positive effect on cell viability, its influence may diminish over time as cells acclimate to the scaffold environment. These findings highlight the potential short-term benefits of PEC in promoting initial cell attachment and proliferation, but they also suggest that for sustained cell viability, additional factors or modifications might be necessary.

The antioxidant activity of PLA_loaded_PEC_sub_ suggests promising results in promoting tissue regeneration and protecting cells from oxidative stress. This result is supported by studies in which similar bioactive compounds showed significant therapeutic effects. For example, a multifunctional DNA hydrogel incorporating procyanidin B2, which has a powerful antibacterial effect against *E. coli* and *S. aureus*, was developed. This hydrogel demonstrated excellent antibacterial properties and promoted tissue regeneration, confirming the potential of integrating bioactive compounds such as procyanidins in hydrogel matrices to enhance their functional properties. The incorporation of procyanidin B2 into the DNA hydrogel highlights the synergistic effects of combining antioxidants with structural biomaterials to create advanced therapeutic systems for wound healing and TE [66].

According to data from the literature, another hydrogel was developed using oligomeric procyanidin, poly(vinyl alcohol), borax, and ferric ions, which demonstrated significant wound healing capabilities. The study concluded that this hydrogel formulation accelerated the wound healing process, likely because of the combined effects of the antioxidant properties of oligomeric procyanidin and the structural support provided by polyvinyl alcohol (PVA) and borax. The inclusion of ferric ions further enhanced the gelation and mechanical properties of the hydrogel, making it a robust and effective material for biomedical applications. These findings are consistent with the results observed in PLA_loaded_PEC_sub_, confirming that the combination of antioxidants with various polymeric systems can lead to multifunctional materials that effectively support and enhance tissue repair and regeneration [67].

### 2.8. Future Directions

Future research on PLA_loaded_PEC_sub_ should focus on conducting in vivo studies and clinical trials to evaluate its biocompatibility, efficacy, and safety in wound healing and tissue regeneration. Exploring different crosslinking methods to develop a gel material, the use of proteins such as gelatin or collagen can enhance the mechanical and biological properties of the scaffold. The development of multifunctional scaffolds that incorporate drug delivery systems and possess antibacterial and anti-inflammatory properties can provide comprehensive solutions for tissue repair and regeneration.

Advanced characterization techniques should be used to gain deeper insights into the microstructure and cellular interactions of the scaffolds, expanding the application of these scaffolds to other types of tissue, such as bone, cartilage, and muscle, can expand their utility in TE [68,69].

## 3. Conclusions

Our study successfully synthesized and characterized novel electrospun PLA fiber scaffolds loaded with encapsulated PEC physical gels. FTIR spectroscopy confirmed the enzymatic polymerization of PEC, and SEM demonstrated the feasibility of producing PLA-loaded PEC scaffolds through electrospinning. The coculture of HDFs with these scaffolds resulted in improved metabolic activity and cell viability, suggesting that PEC-loaded PLA electrospun fiber scaffolds are promising candidates for STE. These findings indicate that these scaffolds could provide significant advantages in regenerative medicine. Future research should focus on conducting in vivo studies and clinical trials to further assess the biocompatibility, efficacy, and safety of these scaffolds in wound healing and tissue regeneration applications. Moreover, exploring different crosslinking methods and developing multifunctional scaffolds with drug delivery systems could enhance their mechanical and biological properties, providing comprehensive solutions for tissue repair and regeneration.

## 4. Materials and Methods

### 4.1. Materials

EC (C_15_H_14_O_6_, ≥90% HPCL), laccase from Trametes versicolor (≥0.5 U/mg), ethyl alcohol (C_2_H_6_O), methanol (CH_3_OH, ≥99.95%), sodium acetate (C_2_H_3_O_2_Na, ≥99.0%), acetic acid (CH_3_OOH, ≥99%), 1, 1, 1, 3, 3,3-hexafluoro-2 propanol (HFP) (C_6_H_5_C(CF_3_)_2_OH, ≥99.0%), N,N-dimethylformamide (C_3_H_7_NO, ≥99.8%), and dichloromethane (CH_2_Cl_2_, ≥99.8%) were purchased from Sigma-Aldrich (St. Louis, MO, USA). PLA (Mw = 230,000 g/mol) was obtained from Nature Works.

### 4.2. Enzymatic Polymerization of EC

The EC monomer is oxidized by laccase to produce PEC. For this purpose, EC (10 mM) was dissolved in a mixture of sodium acetate buffer (0.1 M, pH 5.0) and methanol by magnetic stirring [30]. Two international units [U] per milliliter of laccase were added to the solution, and oxidation was carried out at 60 rpm for 24 h at 50 °C. The total protein concentration of laccase was determined via the Sigma-Aldrich Total Protein Kit [70]. The assay of the enzymatic activity of laccase by oxidation was carried out with ABTS (one unit of laccase [U] = 1 µmol of oxidized ABTS per minute) [71]. The precipitate was recovered by centrifugation at 4500 rpm for 10 min and washed three times with distilled water by centrifugation at 4500 rpm for 10 min. The PEC was dried at 35 °C for 72 h in a vacuum oven.

### 4.3. Emulsion Electrospinning

For electrospinning, PLA has been extensively studied as a biopolymer [72,73,74]. Before being used in electrospinning equipment, PLA underwent a purification process in which the pellets were dissolved in chloroform and then precipitated in ethanol [75]. Previously, solutions of PLA were prepared at concentrations of 6, 13, 16, and 20% *w*/*v* in HFIP. To obtain uniform fibers without defects, the best solution was PLA at a concentration of 20% *w*/*v*. The precipitated PLA was dried at room temperature and then electrospun. For that reason, to obtain uniform fibers, a PLA solution was prepared in HFIP at a concentration of 20% *w*/*v*. The PEC solution was systematically varied: the concentration of (5 and 14% *w*/*v*) in DMF/DCM at a 60:40 ratio, the best condition used to obtain particles, on the basis of their morphology, was 14% *w*/*v*, and the dose used for Xue et al.’s [76] and Ares et al.’s [17] bonding was considered. The electrospinning parameters were 0.3 mL/h, a collector distance of 20 cm, and a potential differential of 16 kV. At the same time, two separate nozzles were used for electrospinning, with one syringe containing the PEC solution and the other containing the PLA solution. The ratio of the PEC and PLA solutions previously described was 1:10.

### 4.4. Fourier Transform Infrared (FTIR) Spectroscopy

FTIR was used to determine the synthesis of PEC using EC as a standard. The samples were analyzed via a Nicolet 6700 (Thermo Fisher Scientific, Waltham, MA, USA). The spectra were scanned in the wavelength range of 4000–500 cm^−1^ at a resolution of 4 cm^−1^.

### 4.5. Contact Angle 

To assess the hydrophobic or hydrophilic properties of the manufactured scaffolds, the contact angle was determined with PBS. Three 1 cm^2^ sections were excised from the scaffolds for use as test samples. Measurements were conducted via a Ramé-hart Model 100-07-00 goniometer (Succasunna, NJ, USA), which was modified with an optical system to precisely capture the interaction between the water droplet and the scaffold surface, and the contact angle was measured after 15 s. ImageJ 1.54g Java 1.8.0_345 software was used to perform a quantitative analysis of the contact angle data. Differences between means were considered not significant (ns) at *p* > 0.05, * *p* ≤ 0.05, ** *p* ≤ 0.01, and *** *p* ≤ 0.001. The results of the analysis are presented as the mean ± standard error of the mean (SEM).

### 4.6. Antioxidant Activity Analysis of EC and PEC

A 7 mM ABTS solution was prepared in water. For the prepared ABTS radical cation (ABTS^•+^), the solution of ABTS was reacted with 2.45 mM potassium persulfate (final concentration) in the dark at room temperature for 12–16 h before use [43].

First, the radical cation (ABTS^•+^) was diluted with 5 mM PBS (pH = 7.4) until the absorbance at 734 nm (A0) was equal to 0.7 ± 0.05. For each 10 μL of the antioxidant compound, 0.9 mL of diluted radical cation (ABTS^•+^) was added, and the absorbance was read at 734 nm after 6 min. The stock solutions of EC and PEC (50–250 μg/mL) in PBS, pH 7.4, were analyzed, and Trolox was used as a control at 50–250 μg/mL. All determinations were carried out in triplicate on a UV-2600 spectrometer (SHIMADZU, Riverwood, MA, USA), and appropriate solvent blanks were used in each assay. The ABTS scavenging activity (%) was calculated via the following equation [35]:

(1) $$ABTS\;scavenging\;activity\left(\%\right)=\frac{A_{0}-A_{1}}{A_{0}}\times 100$$

A_0_ = Absorbance value of the ABTS solution at 734 nm.

A_t_ = Absorbance value of the sample solution at 734 nm.

The results of the analysis are presented as the mean ± standard deviation (SD).

### 4.7. Scanning Electron Microscopy

Electrospun samples with an area of 0.5 cm^2^ were subjected to sputter coating via JEOL JPC-1100 equipment (Tokyo, Japan). The resulting surface morphology was examined using a JEOL field-emitting microscope (JSM-7600F, Tokyo, Japan).

### 4.8. Mechanical Strength Testing 

The tensile test samples were die-cut according to the ASTM D1708 norm, were then tested in a SHIMADZU mechanical testing machine, model AGS-X (Kyoto, Japan). The speed of the test was 10 mm/min. Five samples were tested until rupture. Differences between means were considered not significant (ns) at *p* > 0.05, * *p* ≤ 0.05, ** *p* ≤ 0.01, and *** *p* ≤ 0.001. The results of the analysis are presented as the mean ± standard error of the mean (SEM).

### 4.9. Thermal Assessment

Thermogravimetric analysis (TGA) and differential scanning calorimetry (DSC) were carried out on a TA Instruments DSC Q200 and TGA Q5000IR Module (New Castle, DE, USA). PEC and the scaffold of PLA and PLA_loaded_PEC_sub_ for TGA were measured in a temperature range of 25–600 °C at a heating rate of 10 °C/min in a nitrogen atmosphere. For DSC analysis, the melting temperature, Tm, glass transition temperature, Tg, fusion enthalpy of fusion and crystallization temperature, and Tc were calculated using TA Universal Analysis 5.5 software. DSC was subjected to a heating process from 25 to 300 °C with a temperature increase of 10 °C/min under a nitrogen atmosphere. The thermogravimetric curves were subsequently analyzed using the TA Universal Analysis software.

### 4.10. Cell Viability Assay

Cell viability was assessed through a CCK-8 assay. A total of 1 × 10^4^ cells were seeded in a 96-well plate and subjected to treatment with WST-8 solution at 24, 48, and 72 h. Viability was determined by measuring the absorbance at 460 nm.

Live/Dead^TM^ (calcein/EthD-1) distinguished 7.5 × 104 HDFs in the PLA_loaded_PEC_sub_ after 24 h. The samples were fixed in 2.5% glutaraldehyde, dehydrated in ethanol, and dried using a Samdri-795 Critical Point Dryer (Tousimis, Rockville, MD, USA).

### 4.11. Statistical Analysis

For data analysis, GraphPad Prism was used to perform one-way analysis of variance to compare group means. According to the Tukey test, a *p* value less than 0.05 was considered to indicate statistical significance.

## Data Availability

The data presented in this study are available upon request from the corresponding author.
